# The Effects of Synbiotic “*Bifidobacterium lactis* B94 plus Inulin” Addition on Standard Triple Therapy of* Helicobacter pylori* Eradication in Children

**DOI:** 10.1155/2017/8130596

**Published:** 2017-06-01

**Authors:** Gonca Handan Ustundag, Halime Altuntas, Yasemin Dilek Soysal, Furuzan Kokturk

**Affiliations:** ^1^Faculty of Medicine, Department of Pediatric Gastroenterology, Hepatology and Nutrition, Bulent Ecevit University, Zonguldak, Turkey; ^2^Faculty of Medicine, Department of Pediatrics, Bulent Ecevit University, Zonguldak, Turkey; ^3^Faculty of Medicine, Department of Biostatistics, Bulent Ecevit University, Zonguldak, Turkey

## Abstract

**Aim:**

The aim of this study is to evaluate the effects of the synbiotic* Bifidobacterium lactis* B94 plus inulin addition to the standard triple therapy on* Helicobacter pylori (H. pylori)* infection eradication rates.

**Methods:**

Children aged 6–16 years who had biopsy proven* H. pylori* infection were randomly classified into two groups. The first group received the standard triple therapy consisting of amoxicillin + clarithromycin + omeprazole. The second group was treated with the standard triple therapy and* Bifidobacterium lactis* B94 (5 × 10^9^ CFU/dose) plus inulin (900 mg) for 14 days, concurrently. Eradication was determined by ^14^C-urea breath test 4–6 weeks after therapy discontinuation.

**Results:**

From a total of 69* H. pylori* infected children (F/M = 36/33; mean ± SD = 11.2 ± 3.0 years), eradication was achieved in 20/34 participants in the standard therapy group and 27/35 participants in the synbiotic group. The eradication rates were not significantly different between the standard therapy and the synbiotic groups [intent-to-treat, 58.8% and 77.1%, resp.,* p* = 0.16; per-protocol, 64.5% and 81.8%, resp.,* p* = 0.19]. There was no difference between the groups in terms of symptom relief (*p* = 0.193). The reported side effects were ignorable.

**Conclusion:**

Considering the eradication rates, synbiotic addition to therapy showed no superiority over the standard triple therapy conducted alone. This trial is registered with NCT03165253.

## 1. Introduction

*Helicobacter pylori (H. pylori)* infection is one of the most common chronic bacterial diseases worldwide with an estimated prevalence of more than 50% in adults [1, 2]. Studies in Turkish patients reported that 70–80% of adults and 30–56% of children are infected with* H. pylori* [3–5]. In Western populations,* H. pylori* prevalence is low in children; however, it is still a problem as immigrants from countries where* H. pylori* is endemic constitute a risk group and a reservoir for the infection [6]. If left untreated, it can cause chronic gastritis, peptic ulcer, gastric adenocarcinomas [7], and mucosa-associated lymphoid tissue (MALT) lymphomas [8]. Fortunately,* H. pylori *associated gastric cancer has not been reported in pediatric age group, but MALT lymphomas have been described in a few* H. pylori* infected children [9–11]. In an adult study, early eradication of* H. pylori* has been associated with a sixfold reduction in the recurrence of peptic ulcers as well as a twofold to threefold reduction in the risk of gastric carcinoma [12]. Since* H. pylori* is mostly acquired in childhood, it is important to eradicate the infection in children as well. As stated in Kyoto global consensus report on* H. pylori* gastritis, “the maximum benefit of* H. pylori* eradication is obtained if it is done while the mucosal damage has not progressed beyond the nonatrophic stage” [13]. Further advantages of eradication of the infection in adolescents and young adults are also stated as reduction or prevention of the transmission of the infection to others and especially to their children [13].

Current guidelines recommend eradication of* H. pylori* infection in symptomatic children especially with peptic ulcer disease and with a family history of gastric carcinoma [14, 15]. The standard triple therapy that consists of a proton pump inhibitor and a combination of two antimicrobial agents, amoxicillin and either clarithromycin or an imidazole, for at least 7–14 days, is the main regimen used in both children and adults [14, 16] However, the success rate of this regimen declined from 80 to 96% in the previous years to 60–70% today [17–20]. Sequential therapy or bismuth salts based triple therapies are also mentioned as first-line treatments [14]. With so many different options but far from ideal eradication rates, it is hard to say which treatment is the best for children. Therefore, alternative therapies are still being searched.

Adjuvant treatment with probiotics, prebiotics, and/or synbiotics may be promising options. Probiotics are defined as “living microorganisms that are beneficial to the health of the host when consumed at adequate amounts” [21]. Prebiotics are indigestible foods that enhance the growth or activity of beneficial probiotic bacteria in the gut [22]. Synbiotics are the combination of probiotics and prebiotics, which can work synergistically. In vitro studies demonstrated inhibitory activity of certain probiotic strains against* H. pylori *bacteria [23, 24]. Furthermore, there are randomized controlled trials (RCT) demonstrating favorable outcomes of probiotic addition to* H. pylori *eradication treatments [25–28]. They are believed to increase patient compliance by reducing the side effects such as antibiotic-associated diarrhea [26, 28–30]. Most studies in this area are mainly designed by using a single probiotic strain or a combination of strains. To our knowledge, there are currently a few studies assessing the effects of synbiotics on* H. pylori* eradication [30–32].

In our study, we aimed to investigate the effects of the synbiotic* Bifidobacterium lactis (B. lactis) *B94 plus inulin addition to standard triple therapy in terms of eradication rates, relief of symptoms, and patient compliance.

## 2. Materials and Methods

The study was conducted between June 2011 and June 2012 in the pediatric gastroenterology clinic of Bulent Ecevit University Faculty of Medicine, Zonguldak, Turkey. The study population consisted of 69 children aged 6–16 years who were investigated by a standard esophagogastroduodenoscopy (EGD) for gastrointestinal symptoms, those suggesting an organic disease such as chronic abdominal pain, unexplained nausea and/or vomiting, severe regurgitation, gastrointestinal bleeding, unexplained weight loss, or chronic diarrhea. Written informed consent was obtained from the parents. The study was approved by the Scientific Research Ethics Committee of the Bulent Ecevit University (Protocol number, 2011-10-08/03).

Patients who were treated for* H. pylori *infection previously, who used an antimicrobial agent, bismuth, a nonsteroid anti-inflammatory drug, or any form of gastric acid suppressor during the eight weeks prior the EGD, or who had history of major gastrointestinal surgery, chronic renal, or hepatic disease and who were known to have drug allergy were excluded from the study.

The patients underwent a standard EGD and biopsies from stomach and duodenum were taken at least two per site. Endoscopic findings such as antral hyperemia, antral nodularity, and peptic ulcer were noted. The biopsy specimens were evaluated by the same pathologist at the Department of Medical Pathology, Bulent Ecevit University Faculty of Medicine. Children who were found to have* H. pylori* infection proven by histopathological examination were randomly assigned to two groups in a double blind manner using a prepared randomization list. Each patient was given a number. Patients in the standard triple therapy group were treated with amoxicillin 50 mg/kg/d and clarithromycin 15 mg/kg/d twice daily for 14 days and omeprazole (OMP) 1 mg/kg/d once daily for a month. The synbiotic group received the same standard triple therapy and* Bifidobacterium lactis *B94 (5 × 10^9^ CFU/dose) plus 900 mg of inulin (Maflor sachet, Mamsel, Turkey) given a single dose for 14 days concurrently. Required amount of drugs for both treatment groups was prescribed. A detailed chart explaining the usage of drugs and a form to record the changes in symptoms and the side effects related to treatment (nausea, vomiting, diarrhea, and abdominal or epigastric pain) were given to the patients and/or their parents. The patients were called for follow-up 4–6 weeks after the cessation of OMP treatment. Then, they were assessed for relief of symptoms and ^14^C-urea breath test (^14^C-UBT) was scheduled. Negative ^14^C-UBT was considered as eradication.

## 3. Statistical Analysis

Statistical analysis was performed with SPSS 19.0 software (SPSS Inc., Chicago, IL, USA). Distribution of data was determined by Shapiro-Wilk test. Continuous variables were expressed as mean ± std. deviation, categorical variables as frequency and percent. Continuous variables were compared with the independent sample* t*-test or Mann–Whitney* U* test for two groups. Categorical variables were compared using Pearson's Chi-square test or Fisher Exact Chi-square test. The eradication rates were determined using both the intent-to-treat (ITT) and per-protocol (PP) analyses.* p* value of less than 0.05 was considered statistically significant for all tests.

## 4. Results

**Figure 1:**
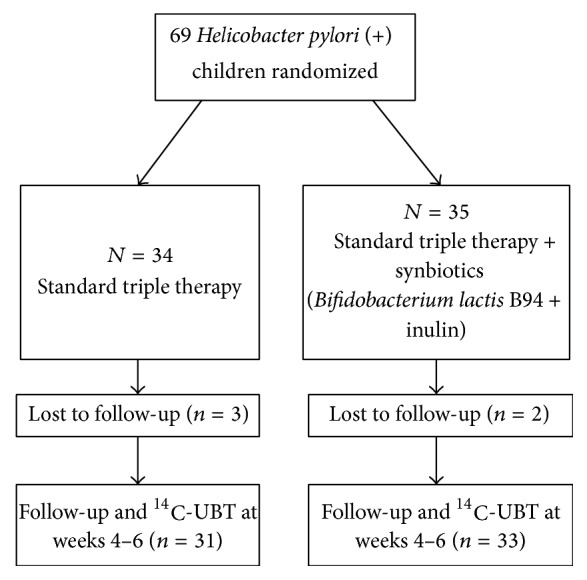
Flow diagram of the patients' progression through the study. ^14^C-UBT: carbon-14 labelled urea breath test.

Sixty-nine patients with* H. pylori* infection were randomly assigned to the standard therapy group (*n* = 34) and synbiotic group (*n* = 35) (Figure 1). The demographic characteristics of patients were similar between the groups (Table 1). The most frequent complaint was abdominal pain in both groups (87% in the standard therapy group and 97% in the synbiotic group,* p* = 0.19). It was mainly described as epigastric pain by 40.7% of patients in the standard therapy group and 43.8% of patients in the synbiotic group (*p* = 0.09). The second frequent complaint was nausea in 64.5% and 60.6% of patients in the standard therapy and synbiotic groups, respectively (*p* = 0.9). A total of 5 children dropped out the study, since they did not present to the follow-up (Table 2). All the other patients completed the treatment as prescribed. Patients were reassessed at follow-up 4–6 six weeks after the end of OMP treatment.

None of the patients in each group experienced a side effect that would require termination of the treatment. Only one patient complained of increased abdominal pain in the standard therapy group, and one patient had a new onset of diarrhea during the antibiotherapy in the synbiotic group. In the standard therapy group, there was no change of symptoms in 4 patients (12.9%), while a slight relief of symptoms in 11 patients (35.5%) and a marked relief of symptoms in 16 patients (51.6%) were observed. In the synbiotic group, no change of symptoms was reported by 1 patient (3%), whereas a slight relief of symptoms and a marked relief of symptoms were reported by 7 patients (21.2%) and by 25 patients (75.8%), respectively. Although, the symptoms were much more improved in the synbiotic group, the difference was not statistically significant between the groups (*p* = 0.09).

*H. pylori* eradication was confirmed by ^14^C-UBT. The eradication rates in both the synbiotic and the standard therapy groups were evaluated using ITT and PP analyses (Table 3). Eradication rates did not differ significantly between the groups (*p* = 0.16 and* p* = 0.19, by ITT and PP analyses, resp.).

## 5. Discussion

In recent years the efficacy of* H. pylori* eradication treatments has fallen below an acceptable level of 80%, mainly due to antimicrobial resistance and inadequate patient compliance [16]. As no new drug has been developed for* H. pylori* eradication, clinical trials have focused on different combinations of known drugs and adjuvant therapy. Probiotics are thought to be beneficial in* H. pylori* infection in two ways: (1) some strains may have a direct effect on the pathogen and inhibit its growth. (2) Some strains may reduce antibiotic-associated side effects and improve compliance to therapy. Moreover, pre- and probiotic supplementation may have immunomodulatory effects on the hosts as experimental studies demonstrated their anti-inflammatory effects in chronic inflammatory diseases like asthma, topical allergies, and inflammatory bowel disease [33, 34].

In vitro studies and animal models have demonstrated inhibitory effects of several probiotic species especially* Lactobacilli *and* Bifidobacterium* on* H. pylori* colonization and growth [23, 24]. This effect is achieved by the production of organic acids, autolysins, mucin, and bacteriocins and/or by the binding of some specific strains to the same glycolipid receptors as* H. pylori* [23, 24, 35–38]. The preclinical studies have supported the possibility of using probiotics in the treatment of* H. pylori* infections in clinical settings.

Several studies evaluated the effects of probiotics as monotherapy in asymptomatic children infected with* H*.* pylori *[31, 39]. Gotteland et al. demonstrated that children receiving* Saccharomyces boulardii (S. boulardii)* and inulin (a synbiotic) had a significant decrease in ^13^C-UBTs [31]. In 12% of the participants, the infection was even eradicated by the regular intake of this synbiotic [31]. Similarly, Cruchet et al. treated* H. pylori* infected asymptomatic children for one month with live* Lactobacillus johnsonii* La 1 and showed significant changes [39]. In summary, specific probiotic strains as mentioned above probably lower the bacterial load in the gastric mucosa but not completely eradicate the bacteria. Considering that a certain percentage of the patients had achieved eradication even with monotherapy, we have planned the current study assuming that we would get better results by combining a synbiotic with standard therapy.

To date, several systematic reviews and meta-analyses have been published about the effects of probiotic supplementation to standard treatment regimens of* H. pylori* infection in both children and adults [40, 41]. The systematic review and meta-analysis by Szajewska et al. evaluated the effects of* S. boulardii* supplementation to eradication therapy [40]. Eleven RCTs involving 2200 patients were included in the review. The eradication rates were found to be significantly higher (80% versus 71%) in participants taking* S. boulardii*. Also, overall adverse effects were lower in the probiotic group compared to the control group, and particularly the risk of diarrhea and nausea was reduced [40]. The recent meta-analysis by Lau et al. included thirty RCTs involving more than 4000 patients, both children and adults [41]. They examined the impact of probiotic supplementation on standard triple therapy and concluded that the addition of probiotics increased eradication rates (*p* < 0.001). Moreover, the risk of diarrhea, nausea, vomiting, and epigastric pain was also found to be reduced. In conclusion, generally the authors suggested that the eradication rates improved, and the frequency of adverse effects was reduced. However, the results should be interpreted cautiously, since collecting data on different strains in meta-analysis may be misleading.

In our study, we evaluated the effects of a synbiotic “*B. lactis *B94 and inulin” addition to standard triple therapy in symptomatic children. Although, the eradication rate seemed to be higher in the synbiotic group, the difference was not statistically significant (ITT,* p* = 0.16, and PP,* p* = 0.19). To our knowledge, there are only two reports that used the same synbiotic treatment in* H. pylori* eradication [30, 32]. Similar to our results, Islek et al. could not find a positive effect of this symbiotic treatment on eradication rates in* H. pylori* infected children [30]. However, there was a notable decrease on the occurrence of side effects in the synbiotic group which was interpreted as an indirect positive effect on eradication success [30]. On the other hand, the study by Çekin et al. revealed significantly higher* H. pylori* eradication rates with sequential treatment and* B. lactis *B94 [32]. Furthermore, they have demonstrated lower diarrhea rates and higher treatment compliance in patients receiving* B. lactis *B94 [32]. Inconsistent with these studies, the patients in our study did not report any side effects that would require discontinuation of therapy. Another recent study by Shafagi et al. also investigated the effectiveness of a multistrain synbiotic combination in adult patients taking quadruple therapy [42]. The eradication rates were higher in the synbiotic group based on ITT analysis (92.1% versus 63.2%,* p* < 0.05) [42]. No significant difference was noted between the groups in terms of side effects. However, a considerable number of patients (14/38) were lost to follow-up in the placebo group, mostly due to noncompliance [42]. The results of this study also showed that compliance is a key factor in eradication success. Indeed, compliance to therapy was not a major problem in our study as none of the patients in both groups had an intolerable side effect. The improvement in dyspeptic symptoms was higher in the synbiotic group, although not statistically significant. However, we did not use an objective symptom scale for this evaluation.

The limitations of our study can be listed as follows: (1)* H. pylori* culture and antibiotic susceptibility tests were not done. (2)* Bifidobacterium* colonization in the feces was not evaluated. (3) The sample size was relatively small.

## 6. Conclusion

The results of our study demonstrated that the addition of* B. lactis *B94 (5 × 10^9^ CFU/dose) plus inulin once daily to standard triple therapy showed no superiority on the eradication rates compared to the standard triple therapy given alone. There are currently a limited number of RCTs in pediatric age group conducted with a diversity of probiotic strains, and the results are conflicting with each other. Thus, it is hard to conclude which probiotic/synbiotic is effective in* H. pylori *eradication, at which doses and duration, and with which antimicrobial regimen. Further large-scale RCTs are needed to clarify these points.

## Figures and Tables

**Table 1:** Demographic characteristics and endoscopic findings of study groups.

	Standard therapy	Synbiotic	
Patient characteristics	*n* = 34	*n* = 35	*p*

Age (year)	11.2 ± 3.1	11.2 ± 2.9	0.97
Sex (female/male)	0.88	1.35	0.55
Height (cm)	145.5 ± 3.0	147.2 ± 3.3	0.71
Weight (kg)	40.9 ± 3.0	42.5 ± 2.5	0.39

Endoscopic findings	*n* = 31	*n* = 33	*p*

Antral hyperemia	26 (83.9%)	28 (84.8%)	0.99
Antral nodularity	4 (12.9%)	4 (12.1%)	
Antral ulcer	1 (3.2 %)	1 (3%)	
Normal duodenum	22 (71%)	27 (81.8%)	0.69
Duodenitis	5 (16.1%)	4 (12.1%)	
Duodenal ulcer	4 (12.9%)	2 (6.1%)	

**Table 2:** The eradication success in study groups.

Status	Standard therapy (*n*)	Synbiotic (*n*)
Eradication	20	27
Failed eradication	11	6
Missing data	3	2

Total	34	35

**Table 3:** The eradication rates in study groups.

Analyses	Standard therapy	Synbiotic	*p*
ITT	56.8% (20/34)	77.1% (27/35)	0.16
PP	64.5% (20/31)	81.8% (27/33)	0.19
